# Performance and Safety of a New Medical Device (Polybactum) for Reducing the Recurrence Rate of Bacterial Vaginosis: Protocol for a Multicenter, Open-Label, Noncontrolled International Clinical Trial (POLARIS Study)

**DOI:** 10.2196/42787

**Published:** 2023-07-20

**Authors:** Filippo Murina, Paolo Inghirami, Marius Biriș, Daniela Sîrbu, Dionisio Franco Barattini, Federica Sbrocca, Luca Ivan Ardolino, Mario Mangrella, Elena Casolati, Serban Roșu, Ciprian Doru Crișan

**Affiliations:** 1 V. Buzzi Hospital University of Milan Milano Italy; 2 AIED Centre Rome Rome Italy; 3 Clinica Medicală Dr Crișan Ciprian Timisoara Romania; 4 Clinica Medicală Dr Sîrbu Daniela Timisoara Romania; 5 Opera Contract Research Organization Timisoara Romania; 6 Italfarmaco Milano Italy; 7 Private Practice of Obstetrics and Gynecology Milano Italy; 8 University of Medicine and Pharmacy “Victor Babeș” Timisoara Romania

**Keywords:** bacterial vaginosis, recurrent bacterial vaginosis, biofilm, polycarbophil

## Abstract

**Background:**

The medical literature has reported that recurrent bacterial vaginosis (RBV) has a relapse rate of 35% within 3 months and 60% within 12 months after antibiotic administration. Products that are able to provide a barrier effect against the biofilm produced by *Gardnerella vaginalis* could play a role in improving the results of bacterial vaginosis (BV) treatment.

**Objective:**

This study aims to assess the performance and safety of a medical device (Polybactum) containing polycarbophil, lauryl glucoside, and glycerides (PLGG) for reducing the rate of recurrence of BV.

**Methods:**

The study includes women who are aged above 18 years, are willing to provide signed informed consent, have a diagnosis of BV according to the Amsel criteria, and have been affected by at least 2 episodes of RBV in the last 12 months. The trial includes 2 phases. In the first phase (according to an open-label noncontrolled design), the treatment involving PLGG is administered for 3 cycles and is followed by a 1-month period of follow-up without treatment. In the second phase, a 9-month follow-up period is envisaged. Thus, for each patient, a 10-month follow-up period without treatment is planned. The study involves 5 centers (2 in Italy and 3 in Romania). We calculated a sample size of 44 pairs to achieve a power of 80% and a 1-sided significance of 5% for detecting a difference of 0.25 between marginal proportions, in comparison with the mean recurrence rate of BV reported in the medical literature. We estimated a potential dropout rate of 20%, and thus, we decided to enroll 55 patients (1-group chi-square test).

**Results:**

The study received ethics approval in 2016 in Romania and 1 year later in Italy. Recruitment started in September 2016. An interim analysis was performed in 2019, and full study analysis results are expected in July 2023.

**Conclusions:**

The tested medical device involving PLGG could modify the mechanisms involved in the pathogenesis of BV and could improve microbiological parameters owing to the acidifying effect on vaginal pH. We believe that the findings of our study could be useful for other investigators who want to test different products against RBV using a standardized protocol and standardized procedures.

**Trial Registration:**

ClinicalTrials.gov NCT02863536; https://clinicaltrials.gov/ct2/show/NCT02863536

**International Registered Report Identifier (IRRID):**

RR1-10.2196/42787

## Introduction

### Overview

The vaginal cavity is colonized in its natural state by Doderleïn bacilli, which include different species of lactobacilli. They form a biofilm over the mucous membrane and hinder the growth, adhesion, and expansion of other potentially pathogenic microorganisms.

These lactobacilli inhibit the growth of pathogens by different means involving several agents [1]. First, lactic acid, produced by the hydrolysis of glycogen contained in vaginal cells, keeps the vaginal pH at a level between 3.8 and 4.5, and thus has a bacteriostatic effect on most vaginal pathogens (excluding *Candida albicans*). Second, hydrogen peroxide, an oxidizing agent secreted by certain lactobacilli (especially *Lactobacillus crispatus* and *Lactobacillus jensenii*) present in 96% of normal vaginal microbiota, hinders the proliferation of anaerobic bacteria [2]. Third, bacteriocins, antimicrobial protein derivatives with a restricted spectrum, act by binding to a specific receptor in the target cell and destabilizing the cytoplasmic membrane through the formation of pores.

Moreover, these lactobacilli produce a barrier against the adhesion of pathogens by means of biosurfactants, which act on surface tension and inhibit the expansion of pathogens through their co-aggregation properties. Bacterial aggregation is a mechanism that leads to the formation of biofilms. Some lactobacilli not only autoaggregate, but can also co-aggregate with pathogenic microorganisms, thus creating a particular microenvironment around the pathogen with significant levels of inhibitors. *Lactobacillus acidophilus*, *Lactobacillus gasseri*, and *Lactobacillus jensenii* co-aggregate with *Candida albicans*, *Escherichia coli*, and *Gardnerella vaginalis*, preventing these pathogenic strains from expanding and adhering to the epithelium [3]. Bacterial vaginosis (BV) is a polymicrobial disease that generally occurs when the protective antibacterial activity of vaginal lactobacilli is impaired, permitting an overgrowth of anaerobes and other pathogens, such as *Gardnerella vaginalis* and *Mycoplasma hominis* [4]. BV occurs in 20% to 25% of the general female population and is the most frequent cause of vaginal discharge [5].

Because of the several risk factors associated with BV, the etiology and pathogenesis of this disease are not completely understood, and the treatment adopted is not always effective, resulting in high recurrence rates [6].

### Rationale

Recurrent bacterial vaginosis (RBV) is defined by 2 to 3 episodes of BV per year [7,8]. The recurrence rates are up to 35% within 3 months [9], 43% to 61% within 6 months, and 49% to 66% within 12 months [10]. It was proven that the tendency to relapse is essentially linked to the capacity of *Gardnerella vaginalis*, and to a lesser extent of *Atopobium vaginae* (recently reclassified as *Fannyhessea vaginae*), to adhere to the mucous membrane, producing a thick biofilm that adheres strongly to the surface of the vaginal mucous membrane [11]. This biofilm is generated within 2 days after the end of standard antibiotic treatment [6]. Thus, several medical reports suggest the use (no later than 24 hours after the discontinuation of antibiotic treatment) of products able to provide a barrier effect against the production of the biofilm by *Gardnerella vaginalis*. Starting from this rationale, it was necessary to find a novel, safe, and effective product able to hinder the formation of this vaginal biofilm, without damaging the fragile equilibrium of the vaginal microbiota.

### Objectives

The primary objective of the study is to evaluate the performance and safety of a 3-cycle treatment of the test product (Polybactum) for reducing the rate of recurrence of BV, considering a 10-month follow-up (ie, without treatment), and compare the obtained results to those reported by appropriately selected international literature.

The secondary objectives are to evaluate the rate of return to normality of the vaginal microflora (defined as vaginal microflora with numerous pleiomorphic lactobacilli, and no other bacteria, clue cells, or hyphae) [12] after the study therapy and to evaluate the safety of the test product.

## Methods

### Overview

The author FM (Milan, Italy) conceptualized the study (ClinicalTrials.gov registration: NCT02863536) and created a team involving a network of gynecologists coordinated by the University of Medicine and Pharmacy “Victor Babeș” (Timisoara, România). The team conducted a systematic review on products already on the market known to be able to provide a barrier effect against biofilm production, assuming that these products, if administered immediately after standard antibiotic therapy, could play a role in improving the results of BV treatment. The literature review established that no gold standard therapy was recommended and that the available treatments were not always effective, resulting in high recurrence rates [4]. In addition, it evidenced, on the one hand, the proven protective activity of these products, and on the other, the low number of clinical trials performed (insufficient to statistically confirm a decrease in the recurrence rate).

In the initial step, the team was involved in building the outline of the protocol and clarifying the yet pending or not identified clinical issues (ie, the product to test and the antibiotic to use). A modified Delphi method [13] was used as a reliable approach to build and create consensus among team attendees. The procedure was defined as follows:

- Polycarbophil would be tested in the planned clinical trial. The characteristic of this well-known product is the formation of a film, which acts as a mechanical barrier able to reduce specific pathogen colonization. The product is already on the market, and the related instruction for use specifies its posology.
- Metronidazole would be the antibiotic therapy to administer following the indication reported in the international guidelines [6]. This drug is the standard treatment for BV at the sites of the involved investigators.
- BV diagnosis would be performed using the Amsel criteria [14], and the Nugent test would be considered as an optional evaluation.
- Patients would be monitored during a 10-month follow-up period (ie, after the end of therapy) to verify the recurrence rate.

The concept synopsis of the planned trial was presented to Effik Italia SpA (Italfarmaco Group, Cinisello Balsamo, Italy) (Multimedia Appendix 1). The company manufactures Polybactum, a medical device containing polycarbophil, lauryl glucoside, and glycerides (PLGG), and it offered the investigator team the medical device and a partial grant to perform the trial on RBV. The agreement between the parties specified that the funder would not have any role in the design and planning of the study, or in the collection, analysis, and interpretation of the data of the future manuscript disclosing the results.

Finally, the team chose the logo (Multimedia Appendix 2), the study name POLARIS (Polybactum to Assess Recurrent Bacterial Vaginosis), and the contract research organization (Opera CRO, a Tigermed Group company, Romania) that would be in charge of the logistics and the management of the trial (protocol submission, project management, site monitoring, data management, and statistical analysis). The coordination of the scientific activity (training of investigators at satellite sites, checking the application of ethics requirements, and circulating scientific information to the study team) would be managed by the Department of Clinical Trials of the University of Medicine and Pharmacy “Victor Babeș” (Timișoara, Romania). By applying this clear division of roles [15], there would be no influence or potential persuasion by the sponsor in the study design, data collection, interpretation, or publication.

### Ethics Approval

The study involved 2 centers in Italy and 3 in Romania. The National Agency for Medicines and Medical Devices of România (*Agentia Nationala a Medicamentului si a Dispozitivelor Medicale*) has approved the study, and the Italian Ministry of Health has received the notification before the start of the trial. The local ethics committees (ECs) in Romania approved the protocol on July 29, 2016 (*EC Clinica Medicală Dr Crișan*; investigator Dr Crișan), October 24, 2016 (*EC Clinica Medicală Dr Crișan*; investigator Dr Biris), and August 22, 2016 (*EC Clinica Medicală Dr Sîrbu*; investigator Dr Sîrbu), and all are located in Timisoara. The formal approvals by Italian ECs were received some months later on December 14, 2016 (#8643/2017; *EC Area 1* in Milan; investigator Dr Murina) and February 15, 2017 (#566/2017; *EC Lazio 1* in Rome; investigator Dr Inghirami) (Multimedia Appendix 3). On March 14, 2018, the study protocol was amended to extend the follow-up period, to allow the calculation (not mandatory) of the Nugent score at baseline and the final visit, and to perform an interim analysis 6 months after the inclusion of the first patient. The results of the interim analysis have been recently published [16].

### Study Design

The trial includes 2 phases. In the first phase (according to an open-label noncontrolled design), the treatment involving PLGG is administered for 3 cycles, and the treatment is followed by a 1-month follow-up. In the second phase, a 9-month follow-up period is envisaged. This entails that for each patient, a 10-month follow-up is planned as follows: 1 month (from the end of the 3rd cycle of PLGG to day 78 [±6 days as the window range]) plus an additional period of 9 months (±2 months as the window range) without treatment from that date to the final visit.

### Study Setting and Patient Recruitment

The 5 centers involved in the study have been selected for their previous large experience in treating BV and for their wide catchment area for patients affected by this infection. The coordinator center is located in Milano (Italy), and the remaining 4 sites are satellite centers (Table 1). In each center, a principal investigator is appointed to be responsible for identification, recruitment, data and sample collection, completion of case report forms, and checking patient adherence to the protocol during the study period.

In addition, each principal investigator is responsible for the transportation of the collected samples to the local laboratory. Indeed, a close relationship between the clinical centers and the laboratories is mandatory to assure optimal performance of the study and to maintain the planned rate of enrollment. Local laboratories analyze each patient’s vaginal smear by phase-contrast optical microscopy. The characteristics of the selected laboratories are reported in Table 2.

Any patient visiting the involved clinical centers during the study period could be enrolled. No flyers, social media, or other advertising methods are used to invite patients to participate in the study.

**Table 1:** Centers involved in the study.

Center type and investigator		Hospital	Town and country
**Coordinator center**			
	Dr Murina	Ospedale Vittore Buzzi	Milano, Italy
**Satellite center**			
	Dr Inghirami	AIED, Via Gorizia	Roma, Italy
	Dr Crișan	Clinica Medicală Dr Crișan Ciprian	Timisoara, Romania
	Dr Biris	Clinica Medicală Dr Crișan Ciprian	Timisoara, Romania
	Dr Sîrbu	Clinica Medicală Dr Sîrbu Daniela	Timisoara, Romania

**Table 2:** Laboratories involved in the study.

Center type and investigator		Hospital	Town and country	Laboratory details
**Coordinator center**				
	Dr Murina	Ospedale Vittore Buzzi	Milano, Italy	The laboratory is inside the center, and the phase-contrast optical microscope is in the same room used for visits.
**Satellite center**				
	Dr Inghirami	AIED, Via Gorizia	Roma, Italy	The laboratory is a private laboratory with extensive experience of collaboration with the lead investigator of the site.
	Dr Crișan	Clinica Medicală Dr Crișan Ciprian	Timisoara, Romania	The center is required to send the samples to a unique specialized laboratory (Bioclinica SA).
	Dr Biris	Clinica Medicală Dr Crișan Ciprian	Timisoara, Romania	The center is required to send the samples to a unique specialized laboratory (Bioclinica SA).
	Dr Sîrbu	Clinica Medicală Dr Sîrbu Daniela	Timisoara, Romania	The center is required to send the samples to a unique specialized laboratory (Bioclinica SA).

### Inclusion Criteria

Patients could enter the study if they fulfilled all of the following criteria: (1) presence of RBV, that is, at least 2 episodes in the last 12 months, including the episode treated before the baseline visit of this study; (2) age above 18 years; (3) BV diagnosis by the Amsel criteria in the 6 to 9 days before the study and cure with metronidazole vaginal formulation (gel for 5 days or ovules for 7 days); (4) no lactation, or lactation and no amenorrhea; and (5) willingness and ability to provide signed informed consent.

### Exclusion Criteria

Patients fulfilling one or more of the following exclusion criteria would be excluded from the study: (1) menstruation at the screening or baseline visit, menopausal transition, or postmenopausal period (2001 STRAW criteria); (2) pregnancy; (3) known allergy to metronidazole or to the tested PLGG ingredients; (4) mixed vaginitis or candidiasis; (5) HIV or other immunodeficiency; (6) sex worker; (7) concomitant enrollment in other interventional clinical trials; (8) unwillingness to provide informed consent; and (9) time between the last day of the last menses and the baseline visit of >16 days or ≤5 days (this criterion was necessary to avoid bias in case of menstrual bleeding occurring during the first PLGG cycle and the consequent need to interrupt the tested product administration).

### Intervention

#### Overview

As specifically requested by ISO 14.155 and MEDDEV 2.12/1 for postmarketing clinical follow-up (PMCF) studies, all the treatments are performed as indicated in the instruction for use for each product.

#### Tested Product

The tested product involving PLGG is a class IIa medical device already on the market as CE QPZ-1805-15 (issue date: March 30, 2015) presented as vaginal ovules. Its components are polycarbophil (Noveon AA1), a barrier-forming agent well known for its lack of toxicity; lauryl glucoside, a nonionic surfactant that enhances barrier properties by reducing surface tension; and Witepsol W35, an administration vehicle. It is a hard, fat suppository base delaying the sedimentation of solid components and promoting the wetting of mucous membranes.

The reasons to choose PLGG for testing in the study are as follows [17]:

- PLGG does not have any effect in terms of general tolerance, because it does not cross the epithelium. Locally, even after prolonged exposure, it does not cause any irritation to the vaginal wall, has no toxic effect on epithelial cells, and does not trigger any sensitivity reaction.
- PLGG has a specific bacteriostatic action that inhibits *Gardnerella vaginalis* growth. In fact, it has been proven that the growth of *Gardnerella vaginalis* was reduced for 48 hours on contact with PLGG (shown already at 24 hours). The inhibitory activity of PLGG has also been demonstrated against *Streptococcus agalactiae* and *Neisseria gonorrhoeae*.
- PLGG has a mucoadhesive property impairing the formation of the biofilm produced by *Gardnerella vaginalis* and allowing the product to stay on the vaginal mucosa for at least 72 hours.
- PLGG has an acidifying effect on vaginal pH, which favors the growth of lactobacillus microbiota and, at the same time, maintains a hostile environment for the recolonization of the vagina by the polymicrobial flora involved in BV.

The mass of the ovule (2.05 g each) ensures coverage of the whole vaginal cavity (about 40 cm^2^).

PLGG vaginal ovule administration is started between 12 and 24 hours after the end of metronidazole vaginal treatment. Each cycle of PLGG has a duration of 1 week as follows: 1 ovule is inserted in the vagina on day 1, 1 ovule is inserted on day 4, and 1 ovule is inserted on day 7. Three cycles of treatment (minimum 72 and maximum 84 days) are required for each patient. At the baseline visit, each patient receives a total of 9 ovules for the whole study duration (3 ovules for 3 cycles). The patient is informed by the investigator to lay in the supine position for some time after ovule insertion.

The baseline visit and the first cycle of PLGG vaginal ovule administration is between 6 and 16 days after menstrual bleeding and after the end of metronidazole treatment. The following warnings are considered:

- If the baseline visit is performed in the morning (and the metronidazole treatment is terminated the previous day), the investigator would give PLGG to the patient to allow PLGG self-administration in the evening.
- If the baseline visit is performed in the evening (and the metronidazole treatment is terminated the previous day), the investigator would deliver PLGG to the patient, permitting PLGG administration directly in the medical office or self-administration at home, in the evening of the same day.
- If the baseline visit is performed in the morning of the same day when the metronidazole treatment is terminated, the investigator would give PLGG to the patient and prescribe PLGG self-administration in the evening with an interval of 12 hours from metronidazole treatment.

In the second and third cycles, PLGG vaginal ovules are administered immediately after the end of the previous menstrual bleeding. For safety reasons, the investigator could stop the administration of PLGG at any time and could prescribe different therapies necessary for the patient’s health.

#### Antibiotic Treatment

Metronidazole (vaginal administration) is the chosen antibiotic therapy (5 g of 0.75% gel once daily for 5 days or 500 mg ovules once daily for 7 days). This drug is largely indicated by the literature as one of the best antibiotic treatments for BV, even if its extensive use and frequent patient nonadherence limit its efficacy in avoiding recurrences [18].

#### Interventions Not Allowed

The following concomitant medications and interventions are not allowed during the study period: oral or vaginal probiotics (eg, vaginal lactobacilli), vaginal tampons, an etonogestrel/ethinyl estradiol vaginal ring (Nuvaring) or intrauterine device, vaginal or oral antibiotic therapy or other vaginal therapies (like spermicide and douching), and other products to treat BV.

### Statistical Methods

#### Power

Data from the medical literature evidenced that the mean recurrence rate of BV after the first episode was from 30% to 50% within 3 months after the appropriate antibiotic therapy [19]. The patients enrolled in our study, after metronidazole treatment, are not allowed to use any vaginal or oral antibiotic therapy. Thus, in this study, it would be realistic to have a mean recurrence rate of 40% after 3 months. Data collected on the recurrence rate after treatment with PLGG vaginal ovules (Effik Italia SpA, unpublished data) showed a 2% correlation between paired observations. We applied a continuity correction, and we obtained a sample size of 44 pairs to achieve a power of 80% and a 1-sided significance of 5% for detecting a difference of 0.25 between marginal proportions. We considered a potential dropout rate of 20%, and thus, we decided to include 55 patients (1-group chi-square test). At 95% CI, a *P* value <.05 is considered statistically significant.

#### Statistical Analysis

The values between visits for the primary objective are analyzed using a *t* test or chi-square test for quantitative variables. In case of binary variables and qualitative variables, a McNemar test and a symmetry test, respectively, are applied. The time to event for BV recurrence is analyzed using Kaplan-Meier curves.

The statistical tests for secondary objectives are chi-square and McNemar tests for categorical data. Moreover, a nonlinear mixed effects model analysis is applied.

All tests are 2-sided, with a nominal level of α=5%. In the primary outcome analysis, *P* values are not adjusted for multiplicity. Statistical analyses are carried out using SAS 9.2 (SAS Institute Inc).

### Evaluation Outcomes

#### Primary Outcome

RBV identified by the Amsel criteria [14] is the primary outcome. The diagnosis requires that 3 of the following 4 criteria are met: (1) vaginal secretion pH greater than pH 4.5; (2) proportion of clue cells ≥20% of total epithelial cells on microscopic examination of the vaginal saline wet mount; (3) off-white and thin vaginal discharge with minimal or absent pruritus and inflammation of the vulva and vagina; and (4) fishy odor on a whiff test.

The Amsel test is considered negative (no diagnosis of BV) when no or only one criterion is met. If 2 criteria are met at baseline, the test is considered unclear, and adopting a conservative approach, the patient is not included. When 2 criteria are met in the final visit, treatment failure is declared.

The Amsel criteria are analyzed by investigators according to the following parameter characteristics:

- Color (off-white, white-grey, or milky) and type (thin and homogeneous) of the vaginal discharge: The investigator performs a direct evaluation of the vaginal secretion.
- Vaginal pH: It is evaluated by investigators using pH paper (Merck) with a range of 4.0 to 7.0. The paper is placed on the lateral vaginal wall to avoid contact with the alkaline secretion of the cervix. Alternatively, pH is evaluated by putting the paper on a swab.
- Whiff test: A sample of the vaginal discharge is placed on a glass slide or in a test tube, and a drop of 10% potassium hydroxide (10% KOH) is added to evaluate the presence of a fishy odor caused by the release of amine.
- Clue cells: Clue cells are checked in local laboratories. A vaginal smear of each patient is collected at the clinical center and sent to the local laboratory. Two drops of vaginal discharge are covered with a coverslip and examined using a phase-contrast optical microscopy technique. Clue cells are identified as vaginal epithelial cells with such a heavy coating of bacteria that the peripheral borders are obscured. The test is positive if microscopic examination of the vaginal saline wet mount shows the presence of clue cells and if the proportion is greater than 20%.

The primary outcome is measured at baseline (day 0), 30 days after discontinuation of PLGG treatment (day 78 [±6 days]), and at the end of follow-up (month 10 [±2 months]). Additional measurements for the primary outcome are performed at unscheduled visits. This is carried out whenever patients report symptoms of RBV to the investigator. The percentage (%) of RBV is calculated considering both the primary outcome results (recurrence of BV identified by means of the Amsel criteria) and the treatment failures reported in the unscheduled visits.

#### Secondary Outcomes

The secondary outcomes are listed and detailed in Table 3.

The secondary outcomes also include the patient global evaluation of performance using a 4-point scale as follows: 1=very good improvement; 2=good improvement; 3=moderate improvement; and 4=negligible improvement.

Safety is evaluated by collecting and analyzing the data on adverse device effects/serious adverse device effects/unanticipated serious adverse device effects and adverse events/serious adverse events during the study period and by globally assessing the safety according to the investigator, using an analogic 4-point scale as follows: 1=excellent; 2=good; 3=fair; and 4=poor.

All secondary endpoints are measured at baseline (day 0) and 30 days after discontinuation of PLGG treatment (day 78 [±6 days]). Only vaginal lactobacilli microbiota is also assessed at the end of follow-up (month 10 [±2 months]).

**Table 3:** Secondary outcome details.

Outcome and score			Evaluation
**Vaginal lactobacilli microbiota**			
	+1 or +2 (normal)	Numerous pleomorphic lactobacilli, and no other bacteria, clue cells, or hyphae. Lactobacillary grade I or IIa.	
	+3 (partially impaired)	Population of lactobacilli is severely decreased. Lactobacillary grade IIb.	
	+4 (pathological)	Lactobacilli are severely depressed or absent because of overgrowth of other bacteria, clue cells (>20%), or hyphae. Lactobacillary grade III.	
**Vaginal discharge (over the last 24 hours)**			
	0	Not present or physiological with regard to quantity, color, and type (white, thin, and flocculent)	
	1	Mild abnormal (abnormal quantity with normal color and type)	
	2	Abnormal quantity, color, and type (white curdy, or thin, greyish, white homogeneous)	
**Burning (over the last 24 hours)**			
	0	Not present	
	1	Mild	
	2	Moderate	
	3	Severe	
	4	Unbearable	
**Erythema**			
	0	No symptoms	
	1	Slight	
	2	Moderate	
	3	Marked	
	4	Very marked	
**Dyspareunia**			
	0	Absent	
	1	Present	

### Study Procedures and Visits

Prior to any procedure of the trial, each patient is informed about the purpose and nature of the study, the potential risks, and the benefits, and is requested to provide consent by signing an informed consent form. Before starting the follow-up period, the patient is required to sign an additional informed consent form detailing the second part of the study.

**Figure 1:**
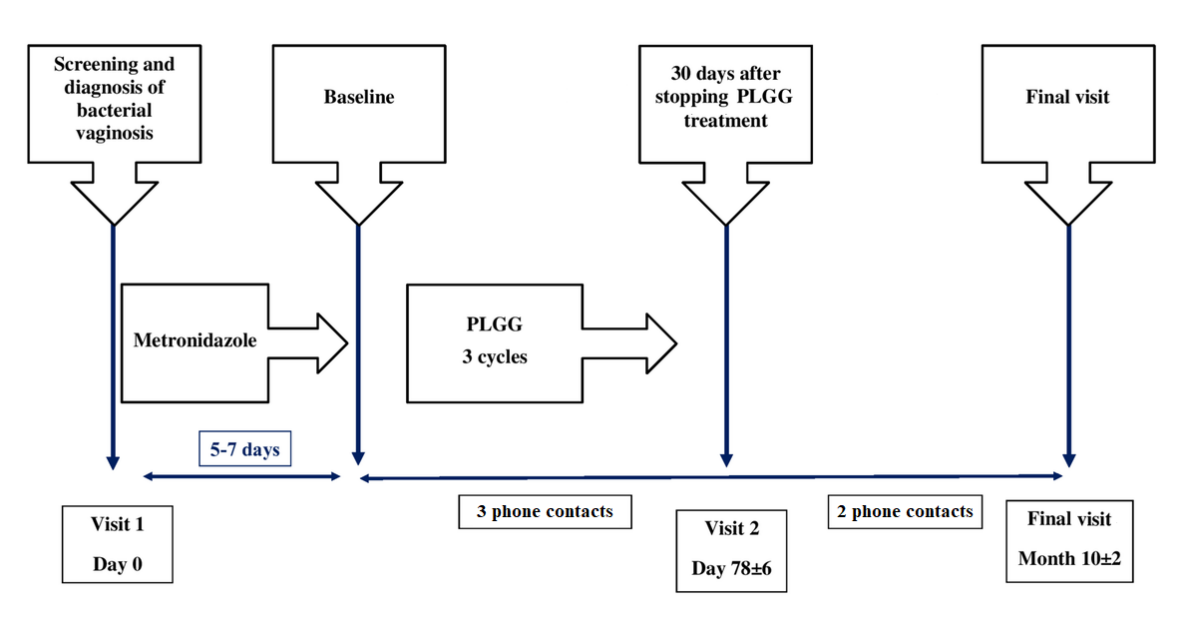
Visits and phone calls planned during the study period. PLGG: polycarbophil, lauryl glucoside, and glycerides.

The visits planned during the study period (Figure 1) are as follows: visit at day 0 (baseline), visit at day 78 (±6 days; 30 days after discontinuation of PLGG treatment), and visit at month 10 (±2 months; end of the follow-up period).

Phone calls with the patient are also planned as follows: first phone contact at 28 (±1) days after the last day of the last menses, second phone contact at 28 (±1) days after the first phone contact, third phone contact at 28 (±1) days after the second phone contact, fourth phone contact at the fourth month of follow-up, and fifth phone contact at the seventh month of follow-up.

During any phone contact, the investigator checks if the patient has BV symptoms, and if present, an unplanned visit is scheduled to confirm the diagnosis of BV based on the Amsel criteria.

The study timeline is presented in Table 4.

**Table 4:** Study timeline.

		Study period							
		Visit 1 (baseline)	Cycle			Visit 2	Month assessment		Final visit
Timepoint		Enrollment (day 0)	1st cycle	2nd cycle	3rd cycle	Day 78±6^a^	Month 4	Month 7	Month 10±2^a^
Eligibility screening		✓							
Medical history		✓							
Informed consent		✓							
Vaginal swab for lactobacilli determination		✓				✓			
**Amsel criteria**									
	Vaginal pH	✓				✓			✓
	Whiff test	✓				✓			✓
	Vaginal discharge	✓				✓			✓
	Clue cells	✓				✓			✓
**Signs and symptoms of BV^b^**									
	Vaginal discharge	✓				✓			
	Burning	✓				✓			
	Erythema	✓				✓			
	Dyspareunia	✓				✓			
Patient global evaluation of performance		✓				✓			
PLGG^c,d^ administration			Within the 6th and 16th day after menstrual bleeding	Immediately after the end of the previous menstrual bleeding	Immediately after the end of the previous menstrual bleeding				
Phone contact			28±1^a^ days after the last day of the last menses	28±1^a^ days after the 1st phone contact	28±1^a^ days after the 2nd phone contact		✓	✓	
Physical examination		✓				✓			
Concomitant medication recording		✓	✓	✓	✓	✓	✓	✓	✓
Check for adverse events		✓	✓	✓	✓	✓	✓	✓	✓
Unused PLGG returned to the investigator						✓			
Diary card delivery		✓				✓			
Diary card collection						✓			✓

^a^Visit window range.

^b^BV: bacterial vaginosis.

^c^PLGG: polycarbophil, lauryl glucoside, and glycerides.

^d^Each cycle of PLGG has a duration of 1 week as follows: 1 ovule is inserted in the vagina on day 1, 1 ovule is inserted on day 4, and 1 ovule is inserted on day 7.

## Results

The recruitment of patients started on September 8, 2016 (first patient and first visit). An interim analysis was conducted in 2019, which has already been published [16], and the results of the full study analysis should be available in July 2023.

## Discussion

*Gardnerella vaginalis* is able to adhere to and displace precoated protective lactobacilli from vaginal epithelial cells, while other BV-associated anaerobes, such as *Fannyhessea vaginae* and *Prevotella*, are less active [20]. Consequently, the current paradigm is that the establishment of the biofilm of *Gardnerella vaginalis* is a required event for the initiation and progression of BV. Since the bacteria within biofilms are not effectively eliminated by the immune system or fully destroyed by antibiotics, biofilm-related infections tend to persist, and thus, not surprisingly, BV tends to have a high rate of relapse and recurrence [5]. PLGG has a specific bacteriostatic action, which inhibits *Gardnerella vaginalis* growth, and its mucoadhesive property could impair the formation of the biofilm produced by these bacteria. The PLGG constituents have important effects on the mechanism involved in the pathogenesis of BV.

In addition, the acidifying effect of PLGG on vaginal pH (favoring the growth of lactobacillus microbiota and maintaining a hostile environment for vagina recolonization by BV polymicrobial flora) could play a key role in improving the microbiological parameters over a long period.

We believe that the findings of our study could be useful for other investigators who want to test different products against RBV using a standardized protocol and standardized procedures. Regarding this, it would be interesting to collaborate with other investigators to test, using the methodology applied in this study, antibiotic therapies indicated in international guidelines [6,21] that are different from metronidazole, as well as other products to replace PLGG. Finally, it could be extremely challenging to conduct our study in geographical regions other than Europe (such as Asia or Africa) or in climatic situations radically different from the continental climate of Romania. In fact, seasonal patterns of abnormal vaginal microbiota have been recently reported in the Burkina Faso population [22], and treatment with oral metronidazole is often ineffective in African women [23].

The absence of an untreated control group represents the main limitation and weakness of the study, but we believe that it is unethical to choose patients experiencing two or more episodes of RBV as an untreated control group. In the near future, when the results of this study are analyzed, we will individuate the proper power of PLGG, and using this information, we will calculate the sample size for a comparative study involving PLGG versus a comparator (ie, a different treatment able to prevent RBV).

The Amsel criteria as originally reported [14] state the presence of clue cells on wet mount microscopy as 1 of the 4 criteria for the diagnosis of BV. The FDA guidelines [24] require not only that clue cells be present, but also that their proportion be greater than 20% of total epithelial cells. This is the clinical practice of the 5 centers involved in the study and is therefore reported in the study protocol, even though it may underestimate RBV cases.

## Appendix

Multimedia Appendix 1 Visual abstract of the POLARIS (Polybactum to Assess Recurrent Bacterial Vaginosis) protocol.

Multimedia Appendix 2 POLARIS logo.

Multimedia Appendix 3 Ethics committee approvals.

## Abbreviations

BV: bacterial vaginosis
EC: ethics committee
PLGG: polycarbophil, lauryl glucoside, and glycerides
RBV: recurrent bacterial vaginosis

## Data Availability

The full protocol of the study has been deposited at protocols.io [25]. In addition, the data sets analyzed during this study are available from the corresponding author on reasonable request.
