# Supplementary material for: Performance and Safety of a New Medical Device (Polybactum) for Reducing the Recurrence Rate of Bacterial Vaginosis: Protocol for a Multicenter, Open-Label, Noncontrolled International Clinical Trial (POLARIS Study)
Source: JMIR Res Protoc. 2023 Jul 20;12:e42787. doi: 10.2196/42787 (PMC10401192; doi:10.2196/42787)
Supplement: Multimedia Appendix 1 [file resprot_v12i1e42787_app1.pdf]

# Testing a new medical device in reducing bacterial vaginosis recurrence (BVR)

35% recurrence rates within 3 months,
50% within 6 months, and 60% within 12 months

BVR identified by Amsel criteria was the primary outcome

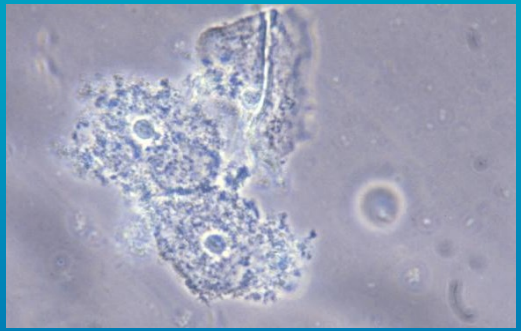

We calculated a sample size of 44 subjects with 80% power and a one-sided significance of 5%; 55 were the enrolled subjects.

Murina F, et al. *JMIR.*

Polaris
MULTINATIONAL CLINICAL TRIAL

**EPICOT model (Brown P, BMJ 2006;333:804–6)**

| | Core Elements | Details of the protocol |
|---|---|---|
| **E** | Evidence | • The systematic reviews do not evidence any treatment able to significantly reduce the recurrence of BV.<br>• Hindering the formation of bad biofilm is a crucial element to assure the positive result of treatment; this could be possible with an early administration of the tested product which has mucoadhesive properties.<br>• Amsel criteria represent the standard to make diagnosis of BV in clinical trials |
| **P** | Population | Women with diagnosis of Recurrent Bacterial Vaginosis (RBV) and cured with vaginal metronidazole in 2 Italian and 3 Romanian Centers (age ≥18 years; diagnosis of RBV by Amsel; non lactating and lactating but only if with menstrual cycle).<br>Women suffering from vaginal candidiasis or mixed infections and women using Nuvaring®) or an intrauterine device were excluded |
| **I** | Intervention | PLGG 3 monthly cycles; administration for each cycle: 1 ovule inserted in vagina at Day 1 after the end of menstrual bleeding; 1 ovule at Day 4; 1 ovule at Day 7. Follow up of 10±2 months |

Murina F, et al. *JMIR.*

EPICOT model (Brown P, BMJ 2006;333:804–6)

| | Core Elements | Details of the protocol |
|---|---|---|
| C | Comparison | No control group for ethic reasons. A comparison with recurrence data reported by selected international literature will be performed |
| O | Outcome | Recurrence of bacterial vaginosis identified by Amsel criteria (vaginal pH, whiff test, homogenous vaginal discharge and clue cells at optical microscopy by phase-contrast;<br>Secondary outcome:<br>Vaginal Lactobacilli microbiota by vaginal swab;<br>Signs and symptoms of BV (vaginal discharge, burning, erythema, itching, dyspareunia)<br>Patient global evaluation of performance<br>Secondary safety outcomes:<br>ADE/SADE/USADE and AE/SAE.<br>Global assessment of safety performed by Investigator |
| T | Time stamp | May 2016 |

Murina F, et al. *JMIR.*

Polaris
MULTINATIONAL
CLINICAL TRIAL

# RBV: 3 monthly cycles of treatment + 10 months follow up

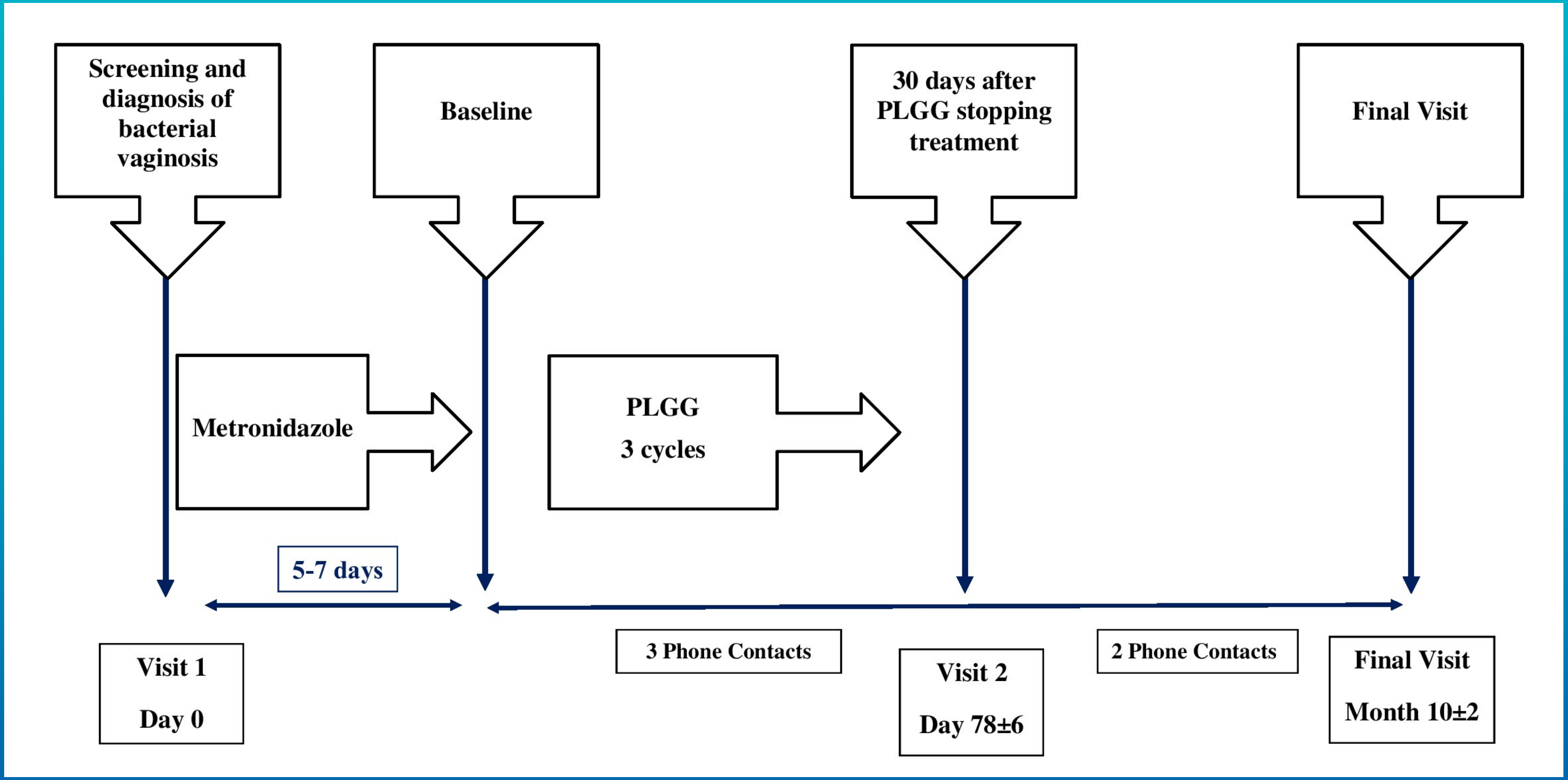


Murina F, et al. *JMIR.*

# The POLARIS study: Protocol for a trial with a 10-month follow up in recurrent bacterial vaginosis

| Study Population | | Intervention | Outcome |
|---|---|---|---|
| **Women affected by RBV, i.e., at least 2 episodes in the last 12 months** | **OPEN STUDY** | PLGG a CE marked medical device containing polycarbophil, lauryl glucoside, glycerides<br>[NON-CONTROLLED<br>3 ovules for 3 cycles of treatment (minimum 72 and maximum 84 days).<br>First cycle after metronidazole therapy | Primary:<br>RBV identified by Amsel criteria<br>Secondary:<br>Vaginal Lactobacilli Microbiota<br>Signs and symptoms of BV<br>Patient performance evaluation<br>Safety:<br>ADE/SADE/USADE<br>AE/SAE<br>Safety global evaluation by Investigator |

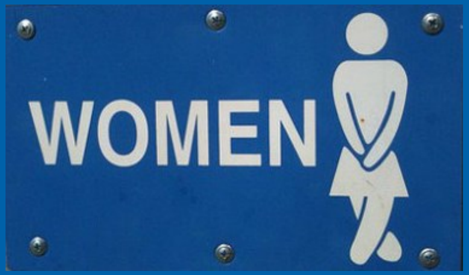


Murina F, et al. *JMIR.*

Polaris
MULTINATIONAL CLINICAL TRIAL

# Study procedures and Visits

**VISITS**

- visit at day 0: baseline
- visit at day 78±6: 30 days after stopping PLGG treatment.
- visit at month 10±2: end of the follow-up period.

- **PHONE CONTACTS**
- 1st phone contact: at 28 ±1 days after the last day of last menses.
- 2nd phone contact: at 28 ±1 days after the 1st phone contact.
- 3rd phone contact: at 28 ±1 days after the 2nd phone contact.
- 4th phone contact: at 4th month of follow up.
- 5th phone contact: at 7th month of follow up.

Murina F, et al. *JMIR.*

Polaris
MULTINATIONAL
CLINICAL TRIAL
