# Supplementary figures and images for: Performance and Safety of a New Medical Device (Polybactum) for Reducing the Recurrence Rate of Bacterial Vaginosis: Protocol for a Multicenter, Open-Label, Noncontrolled International Clinical Trial (POLARIS Study)
Source: JMIR Res Protoc. 2023 Jul 20;12:e42787. doi: 10.2196/42787 (PMC10401192; doi:10.2196/42787)

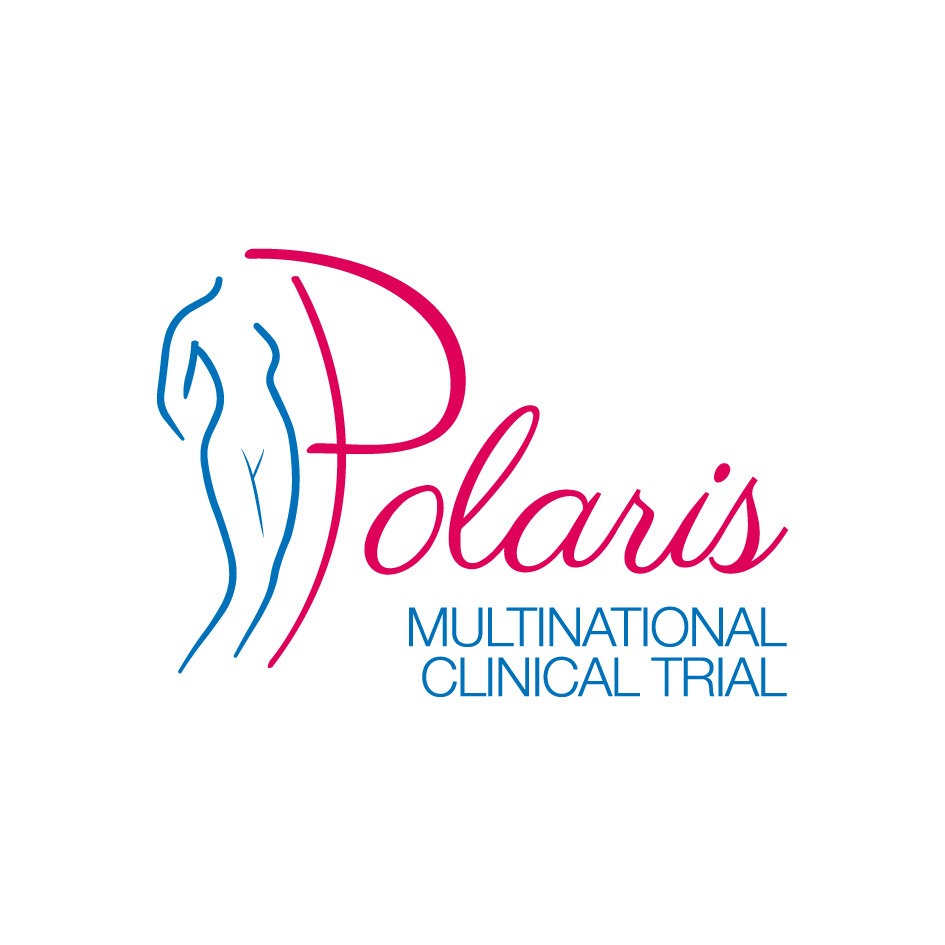

Supplement: Multimedia Appendix 2 [file resprot_v12i1e42787_app2.png]
