# Supplementary material for: Performance and Safety of a New Medical Device (Polybactum) for Reducing the Recurrence Rate of Bacterial Vaginosis: Protocol for a Multicenter, Open-Label, Noncontrolled International Clinical Trial (POLARIS Study)
Source: JMIR Res Protoc. 2023 Jul 20;12:e42787. doi: 10.2196/42787 (PMC10401192; doi:10.2196/42787)
Supplement: Multimedia Appendix 3 [file resprot_v12i1e42787_app3.pdf]

Comitato Etico Milano Area 1
Presidente: Prof.ssa Anna Maria Di Giulio
tel. 02 3904.3518
fax. 02 3904.2030
comitato.etico@asst-fbf-sacco.it

Prot. n° 8643/2017
31/03/2017

Dr. Dionisio Franco Barattini
Medical Director
Opera Contract Research Organization Srl
barattini@operacro.com

Dr. Filippo Murina
ASST Fatebenefratelli Sacco
U.O. Ostetricia e Ginecologia
P.O. Vittore Buzzi
Sede
filippomurina@tin.it

Dr. Carlo Valentini
U.O. Economico Finanziario
Sede
valentini.carlo@asst-fbf-sacco.it

Oggetto: **Protocollo OPEFF/0116/MD**
Studio multicentrico, in aperto e non comparativo della durata di 3 mesi, per valutare la performance e la safety del nuovo dispositivo medico Polybactum® nel ridurre la frequenza della Vaginosi Batterica Ricorrente. (POLARIS: Polybactum® per valutare la Vaginosi Batterica Ricorrente).
Registro Sperimentazioni n. 2016/ST/243.
Approvazione studio.

Si comunica che il Comitato Etico Interaziendale Milano Area A in data 14 dicembre 2016 ha esaminato tutti i documenti trasmessi dalla società OPERA Srl con la lettera datata 29 novembre 2016:

00 Lettera di intenti Milano del 29-09-2016
01a Delega_signed del 21-07-2016
01b Delegation_signed del 21-07-2017
01c Lettera di incarico al PI del 02-11-2016
02 Multinational Protocol final V1 del 04-05-2016
02a firma protocollo coordinatore Dr F Murina
03 Sinossi Italiana final V1 del 04-05-2016
04 Polybactum(R) IB edition n4 del 01-06-2016.
04a Polybactum(R) IB ed n4 Firma
05 Lista CENTRI STUDIO POLARIS versione 1 del 04-05-2016
06 Informativa e consenso informato V1 del 04-05-2016
07 Lettera per medico curante versione 1 del 04-05-2016
08 Trattamento dei dati personali versione 1 del 04-05-2016
09 Flow chart versione 1 del 04-05-2016

10 Polizza certificato EFFIK ITALIA POLARIS del 29-08-2016
10a Insurance Effik Italy-payment
10b assicurazione_pagamento
11 Scatola_E100233A_G4524900_BAG_COM_3 ovuli vta
12 Scheda tecnica e manuale di uso – Polybactum del 05-2015
13 Bozza Convenzione economica
14 CRF POLARIS-final version 1 del 05-05-2016
15 Diario Paziente V1 del 04-05-2016
16 CV Dr. F. Murina
17 Certificato di conformità del 09.03.2016
17a Certificato di fabbricazione_QPZ180515 del 30-03-2015
18 Ricevuta versamento CE
19 Conflitto interessi Dr F Murina
20 Modulo Richiesta parere del Dr F Murina.

Stralcio del Verbale del 14 dicembre 2016

Il Comitato Etico Interaziendale Milano Area A dopo aver valutato la documentazione e sentito il relatore chiede le seguenti le seguenti modifiche :

protocollo
- disegno dello studio: la dimensione del campione non risulta appropriata rispetto all'ipotesi formulata (pazienti con BV dopo tre mesi di trattamento 20%, senza trattamento 40%; cioè dimezzamento dei casi previsti): utilizzando un test di McNemar (Schork, M. and Williams, G. 1980 "Number of Observations Required for the Comparison o Two Correlated Proportions". Comunication in Statistics-Simula. Computa.. B9(4). 349-357) ad una coda e con un errore alfa del 5%, la potenza dello studio , con 43 soggetti valutati, risulta ben inferiore all'80% dichiarato. Definire in modo più dettagliato i metodi utilizzati per il calcolo del sample size e allegare l'output del programma statistico utilizzato per il calcolo.

Foglio informativo
- eliminare l'incongruenza tra ciò che è citato nella sinossi in merito al divieto di utilizzare dispositivi intrauterini rispetto a quanto dichiarato nel foglio informativo in cui, invece, se ne consiglia l'uso;
- eliminare l'interruzione dello studio per motivi amministrativi.

Il Comitato Etico Interaziendale Milano Area A APPROVA lo studio a fronte delle modifiche richieste.

Il Comitato Etico Milano Area 1 in data 22 marzo 2017 prende atto delle modifiche trasmesse dalla società OPERA Srl tramite e-mail del 15 marzo 2017: lettera datata 14 marzo 2017, Foglio Informativo e modulo di consenso versione finale 1.0 del 04.05.2016, Foglio Informativo e modulo di consenso versione finale 2.0 del 14.03.2017, ed approva definitivamente lo studio.

Si raccomanda al fornitore del prodotto oggetto di studio di trasmettere al Servizio di Farmacia dell'ASST Fatebenefratelli Sacco tutto l'eventuale materiale finalizzato allo studio clinico per gli opportuni controlli, la registrazione e la consegna allo sperimentatore.

Si ricorda agli Sperimentatori che il Regolamento del Comitato Etico Interaziendale Milano Area A li obbliga a **compilare il Modulo dello stato di avanzamento dello studio** almeno una volta per studi della durata inferiore ai dodici mesi e almeno una volta all'anno per gli studi di durata superiore.

Si ricorda allo Sperimentatore di notificare la **data di avvio**, successivamente la **data di chiusura** dello studio ed eventuali pubblicazioni scientifiche impattate e non.

Si ricorda allo Sperimentatore Principale che la sperimentazione potrà essere avviata solo a seguito della Delibera autorizzativa da parte del Direttore Generale.

Cordiali saluti.

IL PRESIDENTE
Comitato Etico Milano Area 1
Prof.ssa Anna Maria Di Giulio

Referente Istruttoria: Sig.ra Nicoletta Buonfiglio
Tel. 02 3904 3518

MODULO DI REPORTING SULLO STATO DI AVANZAMENTO DEGLI STUDI CLINICI IN CORSO E CHIUSURA DELLO STUDIO

La compilazione del presente modulo ha lo scopo di comunicare al CE lo stato di avanzamento degli studi clinici in corso presso il centro sperimentatore

Data di rilevazione:	Reparto:	Responsabile della sperimentazione:
Protocollo di studio:<br><br>numero e data Delibera autorizzativa:		Sponsor:

Stato dello studio:
- ☐ In corso (data apertura centro ....)
- ☐ Non iniziato (*)
- ☐ Sospeso (*)
- ☐ Concluso (data chiusura centro ....)
- ☐ Ritirato (*)
- ☐ Interrotto (*)

(*) precisare le motivazioni ....

Farmaci in studio in giacenza in reparto	Lotto	Scadenza

N. totale pz da arruolare:	N. pz arruolati:	N. pz in studio:
N. pz in follow up:	N. pz con trattamento concluso:	N. pz drop out (*):

(*) Motivo per l'interruzione: interruzione volontaria, n. pz:

interruzione per effetti collaterali e/o eventi avversi, n. pz:

altro ______________________________ n. pz:

N. gravidanze durante lo studio:

N. eventi avversi registrati dal centro:	Sono stati segnalati al CE ?	Si	No
	Sono stati segnalati alla Direzione Sanitaria?	Si	No

Tipologia:

Eventuali problemi insorti nel corso della sperimentazione:

Firma del responsabile della sperimentazione:

COMPONENTI IL Comitato Etico Milano Area 1

Istituito con Delibera n. 1224 del 30.12.2016

SEDUTA DEL 22/03/2017 **Numero legale: SI**

ELENCO COMPONENTI E CONSULENTI	PRESENTE
**Prof.ssa Anna Maria Di Giulio**<br>Farmacologo	✓
**Dr. Paolo Borroni**<br>Rappresentante del volontariato o dell'associazionismo di tutela dei pazienti	✓
**Don Tarcisio Bove**<br>Esperto in bioetica	✓
**Dr. Rodolfo Casati**<br>Clinico	✓
**Dr. Raffaele Contini**<br>Medico Medicina Generale	✓
**Dr.ssa Maria Vittoria Cossu**<br>Clinico	✓
**Dr. Giuseppe De Luca**<br>Rappresentante dell'area delle professioni sanitarie interessata alla sperimentazione	✓
**Dr.ssa Gabriella Farina**<br>Clinico	✓
**Dr. Giovanni Guizzetti**<br>Esperto Dispositivi Medici<br>Ingegnere clinico o altra professione qualificata	☐
**Dr.ssa Gaetana Muserra**<br>Farmacista SSR	☐
**Prof. Massimiliano Pagani**<br>Esperto Genetica	☐
**Prof. Antonio Pontiroli**<br>Esperto in Nutrizione	✓
**Dr. Giuliano Rizzardini**<br>Clinico	✓
**Dr. Edoardo Rossi**<br>Esperto Clinico (Tecniche Diagnostiche ecc...)	✓
**Dott. Marco Sala**<br>Pediatra	✓
**Dr.ssa Ida Rosa Salvo**<br>Clinico	✓
**Dr. Carmine Tinelli**<br>Biostatistico	✓
**Prof. Maurizio Viecca**<br>Clinico	☐
**Dr. Antonio Vitello**<br>Medico Legale	✓
**Dr. Tommaso Saporito**<br>Direttore Sanitario ASST Fatebenefratelli Sacco	☐
**Dr. Giampietro Nardi**<br>Sostituto Direttore Sanitario ASST Fatebenefratelli Sacco	✓
**Dr.ssa Mariagrazia Piacenza**<br>Farmacista ASST Fatebenefratelli Sacco	✓
**Dr. Mauro Moreno**<br>Direttore Sanitario ASST Santi Paolo e Carlo	☐
**Dr. Danilo Gariboldi**<br>Sostituto permanente Direttore Sanitario ASST Santi Paolo e Carlo	✓
**Dr.ssa Domenica Di Benedetto**<br>Farmacista ASST Santi Paolo e Carlo	☐

**Dr. Vito Corrao**<br>Direttore Sanitario ASST Melegnano e della Martesana	☐
**Dr.ssa Rita Cursano**<br>Sostituto permanente Direttore Sanitario ASST Melegnano e della Martesana	☐
**Dr. Giuseppe Caravella**<br>Farmacista ASST Melegnano e della Martesana	☐
**Dr. Roberto Riva**<br>Direttore Sanitario ASST Lodi	☐
**Dr.ssa Romana Coccaglio**<br>Sostituto permanente del Direttore Sanitario ASST Lodi	☐
**Dr.ssa Elena Gambarana**<br>Farmacista ASST Lodi	☐

ELENCO CONSULENTI	PRESENTE
Consulente<br>**Dott.ssa Maria Abate**<br>Esperto in materia giuridica e assicurativa	☑
**Dott. Maurizio Gallieni**<br>Esperto in nefrologia	☐

I sopra elencati componenti del Comitato Etico dichiarano che si asterranno dal pronunciarsi su quelle sperimentazioni per le quali possa sussistere un conflitto di interessi di tipo diretto o indiretto.

Comitato Etico Lazio 1

SEGRETERIA TECNICO-SCIENTIFICA
c/o Azienda Ospedaliera San Camillo-Forlanini
E mail: comitatoeticolazio1@scamilloforlanini.rm.it
Sito web: www.comitatoeticolazio1.it

REGIONE LAZIO

Roma, 9 /03/2017
Prot. N. 566 /2017 CE Lazio 1

Dott. Paolo Inghirami
A. I. E. D.
Viale Gorizia 14
Roma 00198
inghiramip@tiscali.it

Dott. Franco Barattini
barattini@operacro.com

Oggetto: parere studio Protocollo OPEFF/0116/MD.

Il Comitato Etico Lazio 1, istituito con deliberazione n. 146 del 12 giugno 2013 della Regione Lazio e costituito con delibera n. 880 del 19 luglio 2013 dall'Azienda Ospedaliera San Camillo- Forlanini, ai sensi del Decreto Legge 13 Settembre 2012 n. 158 e in ottemperanza ai requisiti minimi di cui al Decreto 8 Febbraio 2013 del Ministero della Salute, rinnovato con delibera n. 968 del 4 agosto 2016, si è riunito il giorno 15 Febbraio 2017 alle ore 15,00 presso l'Aula "Giuseppe del Porto" della U.O.C. Laboratorio di Genetica Medica, Padiglione Morgagni 1°P – Ospedale San Camillo - per discutere il seguente ordine del giorno:

- Comunicazioni del Presidente;
- Audizione degli sperimentatori per gli studi di seguito elencati:
  - sperimentazioni cliniche;
  - studi osservazionali;
  - emendamenti ai protocolli di studio;
- Varie ed eventuali.

Sono presenti i seguenti componenti del CE Lazio 1: Prof.ssa Paola Grammatico, , Dr.ssa Diana Giannarelli, Dr.ssa Serena Fattori, Dr.ssa Susanna Ricci, Prof. Cosimo Prantera, Dr. Giovanni Maria Vincentelli, Dr. Francesco Meo, Prof. Francisco Javier Fiz Perez, Prof. Eugenio Donato Di Paola, Dr. Mauro Calvani, Dr. Marco Tubaro, Dr. Alberto Chiriatti, Dr.ssa Anna Ceccorulli.

Assenti giustificati: Prof. Giuseppe Piazza, Dr.ssa Domenica Tassielli, Dr. Marco Montanaro, Dr.ssa Teresa Calamia.

Sono presenti i seguenti componenti della Segreteria Tecnico-Scientifica: Dr.ssa Arianna Pompei, Sig.ra Laura Barberito, Dr. Pier Vittorio Lorizio, Dr.ssa Giorgia Bandiera, Dr.ssa Rita Monterubbianesi.

...omissis...

Il Comitato Etico vista l'istanza del 31/12/2016 della società Opera CRO Srl, pervenuta il 18/01/2017 Prot. n. 67/CE Lazio 1, avente in oggetto l'invio dei documenti relativa allo Studio di cui al Protocollo OPEFF/0116/MD dal titolo "Studio multicentrico, in aperto e non comparativo della durata di 3 mesi, per valutare la perfomance e la safety del nuovo dispositivo medico Polybactum ® nel ridurre la frequenza della Vaginosi Batterica Ricorrente" promosso dalla società Effik Italia Spa e da svolgersi presso il centro A. I. E. D. Associazione Italiana Educazione Demografica con sede in Viale Gorizia 14 - Roma, sotto la responsabilità del Dott. Inghirami Paolo, con allegata la seguente documentazione:

- Lettera di intenti del 05-12-2016
- Delega_signed del 21-07-2016
- Delegation_signed del 21-07-2017
- Dichiarazione di conformità della CRO del 05-12-2016
- Multinational Protocol final V1 del 04-05-2016
- firma protocollo coordinatore Dr F Murina
- firma protocollo Dr. P. Inghirami
- Sinossi Italiana final V1 del 04-05-2016
- Polybactum(R) IB edition n4 del 01-06-2016.
- Polybactum(R) IB ed n4 Firma
- Lista CENTRI STUDIO POLARIS versione 1 del 04-05-2016
- Informativa e consenso informato versione 1 del 04-05-2016
- Lettera per medico curante versione 1 del 04-05-2016
- Trattamento dei dati personali versione 1 del 04-05-2016
- Flow chart versione 1 del 04-05-2016
- Polizza certificato EFFIK ITALIA POLARIS del 29-08-2016
- Insurance Effik Italy-payment
- assicurazione_pagamento
- Scatola_E100233A_G4524900_BAG_COM_3 ovuli vta
- Scheda tecnica e manuale di uso – Polybactum del 05-2015
- Bozza Convenzione economica Clean
- Bozza Convenzione economica TC
- CRF POLARIS-final version 1 del 05-05-2016
- Diario Paziente V1 del 04-05-2016
- CV Dr. P.Inghirami
- Certificato di conformità del 09.03.2016
- Certificato di fabbricazione_QPZ180515 del 30-03-2015
- Ricevuta versamento CE
- Schema presentazione studio Lazio
- Conflitto interessi Dr P. Inghirami
- Richiesta di sperimentazione_Dr Inghirami
- Parere unico
- 1 copia cartacea della documentazione

- 1 CD-ROM per il Comitato Etico

Il Comitato etico, al termine della discussione, verificata l'istanza del 31/12/2016 della società Opera CRO Srl, pervenuta il 18/01/2017 Prot. n. 67/CE Lazio 1 e l'allegata documentazione , all'unanimità esprime parere favorevole sullo studio di cui al Protocollo OPEFF/0116/MD promosso dalla società Effik Italia Spa e da svolgersi presso il centro AIED - Viale Gorizia 14 - Roma, sotto la responsabilità del Dott. Inghirami Paolo.

Il Presidente del Comitato Etico Lazio 1
Prof.ssa Paola Grammatico

Clinica Medicală Dr. SÎRBU DANIELA
Timişoara, Str. Apostol Petru şi Pavel, nr. 15

Decizia Comisiei Locale de Etică pentru cercetare ştiinţifică a
Clinica Medicală Dr. SÎRBU DANIELA

Ca răspuns la adresa Dumneavoastră, vă comunicam avizarea din punct de vedere etic al studiului clinic intitulat:

Studiu multicentric, deschis, noncomparativ cu durata de 3 luni pentru evaluarea performanţei şi siguranţei noului dispozitivul medical POLYBACTUM® în reducerea frecvenţei vaginitei bacteriene recurente

Investigator Principal: Dr. DANIELA SÎRBU

Comisia Locală de Etică pentru cercetare ştiinţifică a Centrului Clinica Medicala Dr. SÎRBU DANIELA funcţionează în conformitate cu prevederile art. 167 din Legea nr. 95/2006, art. 28, cap. VIII din ordinul 904/2006, Directivei 2001/20/EC A Parlamentului European şi a consiliului din 4 aprilie 2001 şi cu Declaraţia de la Helsinki –editia 64th emisă de OMS la Fortalezza, Brazilia, Octombrie 2013.

În urma analizei documentelor transmise, Comisia de Etică avizează favorabil desfaşurarea studiului clinic sus-mentionat.

Cu stimă,

Dr. Cernescu Catalin - Preşedinte Comisia de Etică

Dr. Herteg Dorina - Membru Comisia de Etică

Uşvad Selena - Membru Comisia de Etică

Pater Flavius - Membru Comisia de Etică

Macovei Alina - Membru Comisia de Etică

Timişoara, Data 22.08.2016

PFA Biris Marius – Medic Independent
Timişoara, Str . Salcâmilor nr. 22

Decizia Comisiei Locale de Etică pentru cercetare ştiintifică a Centrului ***PFA Biris Marius – Medic Independent***

Ca răspuns la adresa Dumneavoastră, vă comunicam avizarea din punct de vedere etic al studiului clinic intitulat:

Studiu multicentric, deschis, noncomparativ cu durata de 3 luni pentru evaluarea performanţei şi siguranţei noului dispozitivul medical POLYBACTUM® în reducerea frecvenţei vaginitei bacteriene recurente

Investigator Principal: ***PFA Biris Marius – Medic Independent***

Comisia Locală de Etică pentru cercetare ştiinţifică a Centrului Clinica Medicala Dr. Crişan Ciprian funcţionează în conformitate cu prevederile art. 167 din Legea nr. 95/2006, art. 28, cap. VIII din ordinul 904/2006, Directivei 2001/20/EC A Parlamentului European şi a consiliului din 4 aprilie 2001 şi cu Declaraţia de la Helsinki –editia 64th emisă de OMS la Fortalezza, Brazilia, Octombrie 2013.

În urma analizei documentelor transmise, Comisia de Etică avizează favorabil desfaşurarea studiului clinic sus-mentionat.

Cu stimă,

Dr. Costea Daniel - Preşedinte Comisia de Etică

Dr. Fraţilă Mihaela - Membru Comisia de Etică

Militaru Ciprian - Membru Comisia de Etică

Av. Bizera Ruxandra - Membru Comisia de Etică

Petcu Florina - Membru Comisia de Etică Petcu

Timişoara,

Data 24.10.16.

Clinica Medicală Dr. CRIȘAN CIPRIAN
Timișoara, Str . Salcâmilor nr. 22

Decizia Comisiei Locale de Etică pentru cercetare științifică a Centrului **Clinica Medicală**

Dr. CRIȘAN CIPRIAN

Ca răspuns la adresa Dumneavoastră, vă comunicam avizarea din punct de vedere etic al studiului clinic intitulat:

Studiu multicentric, deschis, noncomparativ cu durata de 3 luni pentru evaluarea performanței și siguranței noului dispozitivul medical POLYBACTUM® în reducerea frecvenței vaginitei bacteriene recurente

Investigator Principal: Dr. Ciprian Crișan

Comisia Locală de Etică pentru cercetare științifică a Centrului Clinica Medicala Dr. Crișan Ciprian funcționează în conformitate cu prevederile art. 167 din Legea nr. 95/2006, art. 28, cap. VIII din ordinul 904/2006, Directivei 2001/20/EC A Parlamentului European și a consiliului din 4 aprilie 2001 și cu Declarația de la Helsinki –editia 64th emisă de OMS la Fortalezza, Brazilia, Octombrie 2013.

În urma analizei documentelor transmise, Comisia de Etică avizează favorabil desfașurarea studiului clinic sus-mentionat.

Cu stimă,

Dr. Costea Daniel - Președinte Comisia de Etică

Dr. Frațilă Mihaela - Membru Comisia de Etică

Militaru Ciprian - Membru Comisia de Etică

Av. Bizera Ruxandra - Membru Comisia de Etică

Petcu Florina - Membru Comisia de Etică

Timișoara, Data ...29.07.16

Clinica Medicală Dr. Crişan Ciprian
Timişoara, Str . Salcâmilor nr. 22

Decizia Comisiei Locale de Etică pentru cercetare ştiintifică a Centrului ***Clinica Medicală Dr. Crişan Ciprian***

Ca răspuns la adresa Dumneavoastră, vă comunicăm că am analizat modificările incluse in protocolul 2.0 datat 01.03.2018 pentru studiul cu titlul:

Studiu multicentric, deschis, noncomparativ cu durata de 3 luni pentru evaluarea performanţei şi siguranţei noului dispozitivul medical POLYBACTUM® în reducerea frecvenţei vaginitei bacteriene recurente

Investigator Principal: ***Dr. Doru Ciprian Crişan***

Comisia Locală de Etică pentru cercetare ştiinţifică a Centrului Clinica Medicala Dr. Crişan Ciprian funcţionează în conformitate cu prevederile art. 167 din Legea nr. 95/2006, art. 28, cap. VIII din ordinul 904/2006, Directivei 2001/20/EC A Parlamentului European şi a consiliului din 4 aprilie 2001 şi cu Declaraţia de la Helsinki –editia 64th emisă de OMS la Fortalezza, Brazilia, Octombrie 2013.

În urma analizei documentelor transmise, Comisia de Etică avizează favorabil desfaşurarea studiului clinic sus-mentionat.

Cu stimă,

Dr. Costea Daniel - Preşedinte Comisia de Etică

Dr. Fraţilă Mihaela - Membru Comisia de Etică

Militaru Ciprian - Membru Comisia de Etică

Av. Bizera Ruxandra - Membru Comisia de Etică

Petcu Florina - Membru Comisia de Etică

Timişoara, Data 12.03.2018

Clinica Medicală Dr. Sîrbu Daniela
Timişoara, Str . Apostol Petru şi Pavel, nr. 15

Decizia Comisiei Locale de Etică pentru cercetare științifică a Centrului ***Clinica Medicală Dr. Sîrbu Daniela***

Ca răspuns la adresa Dumneavoastră, vă comunicăm că am analizat modificările incluse in protocolul 2.0 datat 01.03.2018 pentru studiul cu titlul:

Studiu multicentric, deschis, noncomparativ cu durata de 3 luni pentru evaluarea performanţei şi siguranţei noului dispozitivul medical POLYBACTUM® în reducerea frecvenţei vaginitei bacteriene recurente

Investigator Principal: ***Dr. Daniela Teodora Sirbu***

Comisia Locală de Etică pentru cercetare științifică a Centrului ***Clinica Medicală Dr. Sîrbu Daniela*** funcționează în conformitate cu prevederile art. 167 din Legea nr. 95/2006, art. 28, cap. VIII din ordinul 904/2006, Directivei 2001/20/EC A Parlamentului European și a consiliului din 4 aprilie 2001 și cu Declarația de la Helsinki –editia 64th emisă de OMS la Fortalezza, Brazilia, Octombrie 2013.

În urma analizei documentelor transmise, Comisia de Etică avizează favorabil desfașurarea studiului clinic sus-mentionat.

Cu stimă,

Dr. Cernescu Catalin - Preşedinte Comisia de Etică

Dr. Herteg Dorina - Membru Comisia de Etică

Uşvad Selena - Membru Comisia de Etică

Pater Flavius - Membru Comisia de Etică

Macovei Alina - Membru Comisia de Etică

Timişoara,

Data 12.03.2018

PFA Biris Marius – Medic Independent
Timişoara, Str . Salcâmilor nr. 22

Decizia Comisiei Locale de Etică pentru cercetare ştiintifică a Centrului CLINICA MEDICALĂ DR. CRIŞAN CIPRIAN

Ca răspuns la adresa Dumneavoastră, vă comunicăm că am analizat modificările incluse in protocolul 2.0 datat 01.03.2018 pentru studiul cu titlul:

Studiu multicentric, deschis, noncomparativ cu durata de 3 luni pentru evaluarea performanţei şi siguranţei noului dispozitivul medical POLYBACTUM® în reducerea frecvenţei vaginitei bacteriene recurente

Investigator Principal: ***Dr. Biris Marius***

Comisia Locală de Etică pentru cercetare ştiinţifică a Centrului Clinica Medicala Dr. Crişan Ciprian funcţionează în conformitate cu prevederile art. 167 din Legea nr. 95/2006, art. 28, cap. VIII din ordinul 904/2006, Directivei 2001/20/EC A Parlamentului European şi a consiliului din 4 aprilie 2001 şi cu Declaraţia de la Helsinki –editia 64th emisă de OMS la Fortalezza, Brazilia, Octombrie 2013.

În urma analizei documentelor transmise, Comisia de Etică avizează favorabil desfaşurarea studiului clinic sus-mentionat.

Cu stimă,

Dr. Costea Daniel - Preşedinte Comisia de Etică

Dr. Fraţilă Mihaela - Membru Comisia de Etică

Militaru Ciprian - Membru Comisia de Etică

Av. Bizera Ruxandra - Membru Comisia de Etică

Petcu Florina - Membru Comisia de Etică

Timişoara, Data 14.03.2018
